# Promoting migrant HL, good practice in adult education and lessons learned for other interventions

**DOI:** 10.1093/eurpub/ckac131.357

**Published:** 2022-10-25

**Authors:** S Harsch

**Affiliations:** University of Education Freiburg, Freiburg, Germany

## Abstract

**Problem:**

Migration is a global phenomenon, and migrants face myriad challenges, e.g., building context-specific health literacy (HL). To sustainably promote HL, translations, interpreters, or programs in other languages are insufficient. Courses that promote HL holistically are needed, e.g., second language courses. In the SCURA research project, part of the HLCA Consortium, we ethnographically studied language courses and developed interventions. The insights gained are relevant not only for courses promoting HL of migrants or in Germany but also for other target groups and countries.

**Description:**

Based on extensive ethnographic research, we participatory created interventions to promote HL. As language courses promote HL to varying degrees but are severely limited by the rigid conditions and support, we identified strategies to improve HL therein and ensure uptake and sustainability: add-in, pimp-up, dive deeper. The multimodal intervention consists of an extensive collection of teaching ideas, materials to prepare and reflect on sessions, and a 6-part teacher training (in-house, online, and self-study course, based on adult learning principles). The 90-minutes sessions address Health in Language Courses, Health and Me, Using materials, Critically Analyzing Materials and Developing Empowering Activities, Promoting Family HL, and Mental HL. A hands-on tool was developed to help teachers intentionally promote the seven components of HL.

**Results:**

The preliminary results of the ongoing evaluation showed that the flexibly adaptable and applicable offerings, the online workshop, and self-study courses, were well received. The teachers liked the choice of topics and the combination of short inputs and many recommendations for practice.

**Lessons:**

The project’s success relies on knowing the context and setting, considering the needs of all stakeholders, and developing offerings that are a relief but not an additional burden, and that can be easily integrated into the program.

**Key messages:**

• A thorough ethnographic understanding of the course is key to developing interventions that will be perceived to be appropriate and relevant.

• HL promotion should integrate teachers’ HL, informal occasions on health information exchange, diverse and multilingual ways to engage with health information and a systematic, deliberate development.

